# “A novel highly stable and injectable hydrogel based on a conformationally restricted ultrashort peptide”

**DOI:** 10.1038/srep31167

**Published:** 2016-08-10

**Authors:** Chaitanya Kumar Thota, Nitin Yadav, Virander Singh Chauhan

**Affiliations:** 1 International Centre for Genetic Engineering & Biotechnology Aruna Asaf Ali Marg, New Delhi-110067, India

## Abstract

Nanostructures including hydrogels based on peptides containing non protein amino acids are being considered as platform for drug delivery because of their inherent biocompatibility and additional proteolytic stability. Here we describe instantaneous self-assembly of a conformationally restricted dipeptide, LeuΔPhe, containing an α,β-dehydrophenylalanine residue into a highly stable and mechanically strong hydrogel, under mild physiological aqueous conditions. The gel successfully entrapped several hydrophobic and hydrophilic drug molecules and released them in a controlled manner. LeuΔPhe was highly biocompatible and easily injectable. Administration of an antineoplastic drug entrapped in the gel in tumor bearing mice significantly controlled growth of tumors. These characteristics make LeuΔPhe an attractive candidate for further development as a delivery platform for various biomedical applications.

Molecular self-assembly involves many non-covalent interactions with which basic molecular building blocks organize spontaneously and reversibly into novel, supramolecular, functional nano-scale architectures. This bottom-up approach is being used in fabricating various nanostructures for potential applications in biomedical, electronic and optical fields^1^. Self-assembly is also used in generation of hydrogels which are three dimensional (3D) networks of fibrilar chains that retain large amount (>90%) of water and are composed of either high molecular weight natural biopolymers such as polypeptides and polysaccharides or synthetic polymers such as poly(vinyl alcohol), poly(acrylic acid) and poly(ethylene oxide)^2^. Novel approaches in polymer design have revolutionized the field of biocompatible hydrogels. Self-assembly of these polymers into hydrogels under physiologically compatible conditions provides opportunities to develop efficient drug delivery system for wide range of biologically active compounds. Their characteristic properties like high water content, high mechanical strength, biodegradability and biocompatibility make them suitable for potential applications in drug delivery as an encapsulating matrix, as scaffold for 3D growth of cells, wound dressing, synthetic extracellular matrix, implantable devices, biosensors, microfluidics and hygiene products^3^. A number of hydrogels are under investigation and in clinical trials. However, most hydrogels described in literature have a few inherent limitations such as their poor mechanical strength (~2 kPa for gelatine hydrogel)^4^, relatively low homogeneity, poor loading of hydrophobic drugs and lack of biodegradability and biocompatibility which need to be addressed before their effective and safe human use.

Self-assembling peptides serves as attractive candidate for the development of hydrogels with well-controlled biological, mechanical and material properties. Peptide based hydrogels offer several advantages such as their easy synthesis, characterization and decoration, biodegradability and most importantly their very high biocompatibility^5^. Recent findings that relatively short peptides (di-, tri- and tetra-peptides) can readily self-assemble into ordered nanostructures including hydrogels, have made this area of research very active and exciting^6^,^7^.

Here, we report spontaneous self-assembly of a dipeptide, Leucine-α, β-dehydrophenylalanine, containing a non-protein amino acid, α,β-dehydrophenylalanine (ΔPhe) at its C-terminal, into a highly stable hydrogel under physiological conditions. ΔPhe is an analogue of phenylalanine, with a double bond between C_α_ and C_β_ atoms, whose incorporation in peptide sequences introduces conformational constraint in the peptide backbone and provides increased resistance to enzymatic degradation^8^,^9^,^10^. The hydrogel formed by LeuΔPhe was translucent, self-supportive, fractaline in nature, of high mechanical strength, non-toxic, injectable, proteolytically stable and responsive to external stimuli like ionic strength, pH and temperature. Fibrilar network of the dipeptide gel could encapsulate and release various hydrophobic and hydrophilic drug molecules in a controlled manner. The gel restored its original strength after disruption of its structure, indicating its thixotropic behaviour. Administration of the antineoplastic drug, mitoxantrone, entrapped in LeuΔPhe hydrogel in tumor bearing mice, significantly controlled growth of tumors and enhanced the antitumor activity of the drug. These unique characteristics of this low molecular weight dipeptide hydrogel make it an exciting candidate for further development as a drug delivery platform.

## Results and Discussion

**Figure 1:**
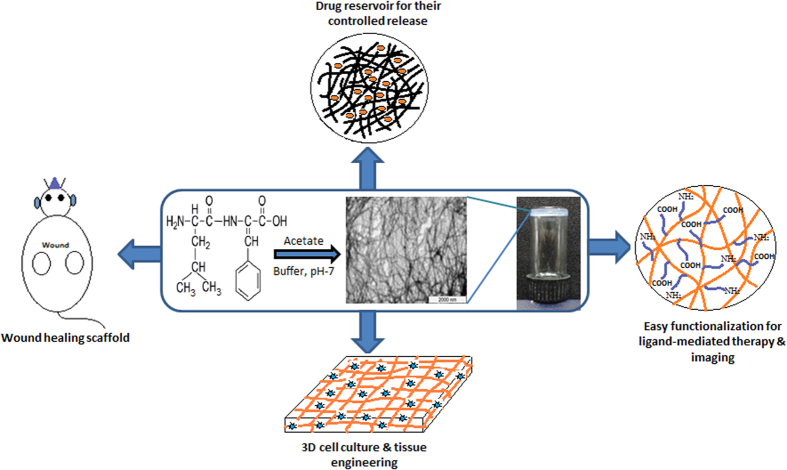
Instant self-assembly of LeuΔPhe into a highly stable hydrogel at room temperature and its possible applications in biomedical field.

**Figure 2:**
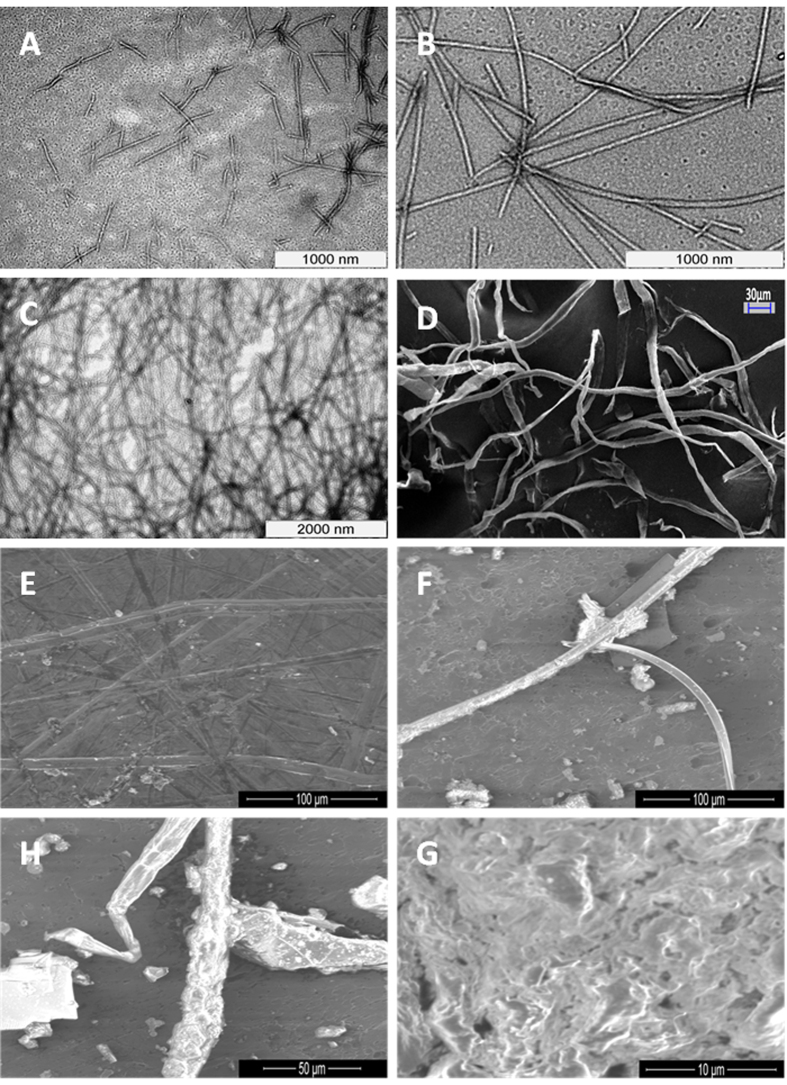
Electron micrographs of Dp gel at different concentrations. **(A)** TEM image of 0.3wt% Dp gel showing small fibrils of >100 nm length. **(B)** TEM image of 0.4wt% LeuΔPhe showing relatively longer and branched fibrils of length in micrometers. **(C)** TEM image of 0.5wt% Dp gel indicating presence of dense network of fibrils. **(D)** SEM image of 0.5wt% LeuΔPhe showing dense fibrilar network of the gel. **(E–H)** ESEM images showing porous fibrilar mesh of 0.5wt% Dp gel.

We synthesized and investigated self-assembly of a large panel of peptides (Supplementary information Table-S1), all containing a C-terminal ΔPhe and found that only one, LeuΔPhe self-assembled instantaneously in 0.8 M sodium acetate buffer pH-7 into a strong hydrogel at room temperature (Fig. 1). Hydrogel formed by LeuΔPhe (Dp gel) was self-supporting, colourless and translucent. DLS experiment with increasing peptide concentration showed an increase in size of the nanostructures. However, beyond 0.4wt% DLS could not be carried out due to increase in size of self-assembled structures (~2 μm) (Supplementary information Figure S-1A). Thio T experiment was also carried out in the same range (0.05wt–0.4wt%), which also supported the DLS results (Supplementary information Figure S-1B). Turbidity measurement experiment^11^,^12^ carried out for gel formation kinetics did not show significant changes till 0.4wt%. However beyond this concentration there was a steep increase in turbidity, indicating formation of dense fibrilar structures at higher concentrations (Supplementary information Figure S-2). TEM showed a distinct increase in self-assembly with increase in concentration. At 0.3wt%, fibrils of >100 nm in length were observed (Fig. 2A). Length of fibrils increased to micrometers along with branching at 0.4wt% (Fig. 2B) which suggested the fibrilar mesh to be the structural basis of the gel^13^. A highly dense network of fibrils was observed in 0.5wt% Dp gel (Fig. 2C), which was also supported by SEM (Fig. 2D). Morphology of Dp gel was investigated using environmental scanning electron microscopy (ESEM) and we found that the fibrils formed by Leu∆Phe exhibited long tubular structures with porous sponge like surface morphologies (Fig. 2E–H).

Molecular structure of the gel was investigated using CD and FTIR spectroscopy. CD spectrum of Dp gel showed a large negative peak at ~202 nm and a strong positive peak at 229 nm, representing π-π* transition and n-π* transitions respectively, indicating the presence of a mixture of β-like as well as extended structures^14^. The positive peak at 229 nm is also reported to represent interactions between aromatic chromophres like phenylalanine in peptides^15^. The strong negative peak observed at 275 nm can be assigned to excitation splitting of the electronic transition of ΔPhe chromophore, probably from a rigid mutual disposition of two or more ΔPhe residues (Supplementary information Figure S-3A). This type of splitting also represents a system of consecutive β-turns^16^. IR spectrum of the gel showed a small characteristic peak at 1645 cm^−1^ (amide I) and another strong peak at 1579 cm^−1^ (amide II). The position of amide I band supported the presence of β-like structure in the gel whereas the band at 3489 cm^−1^ was suggestive of extensive hydrogen bonding in the peptide backbone (Supplementary information Figure S-3B)^17^,^18^.

Leu-Phe, a natural analogue and IIeΔPhe, an isomeric peptide of LeuΔPhe did not form hydrogel in 0.8 M sodium acetate buffer pH-7 (data not shown). Evidently, conformational constraints brought in by the presence of ΔPhe in both, the peptide backbone as well as in the side chain, have assisted in gel formation likely through enhanced π-π* interactions due to extended stacking. This clearly underscores that the small but subtle changes in structure can significantly alter the outcome and nature of the self-assembly of a peptide (Supplementary information Figure S-4)^8^,^9^. Instantaneous gel formation by LeuΔPhe, with both the N- and C- terminals free, is exceptional in comparison to other short peptide based hydrogels described in the literature^7^. For example, a dipeptide like Fmoc-FF also gelates but has its N-terminal protected with a large aromatic protecting group which plays a crucial role in gelation, as also in case of single Fmoc protected amino acids (Fmoc-F, Fmoc-T) that can form gels^19^,^20^.

**Figure 3:**
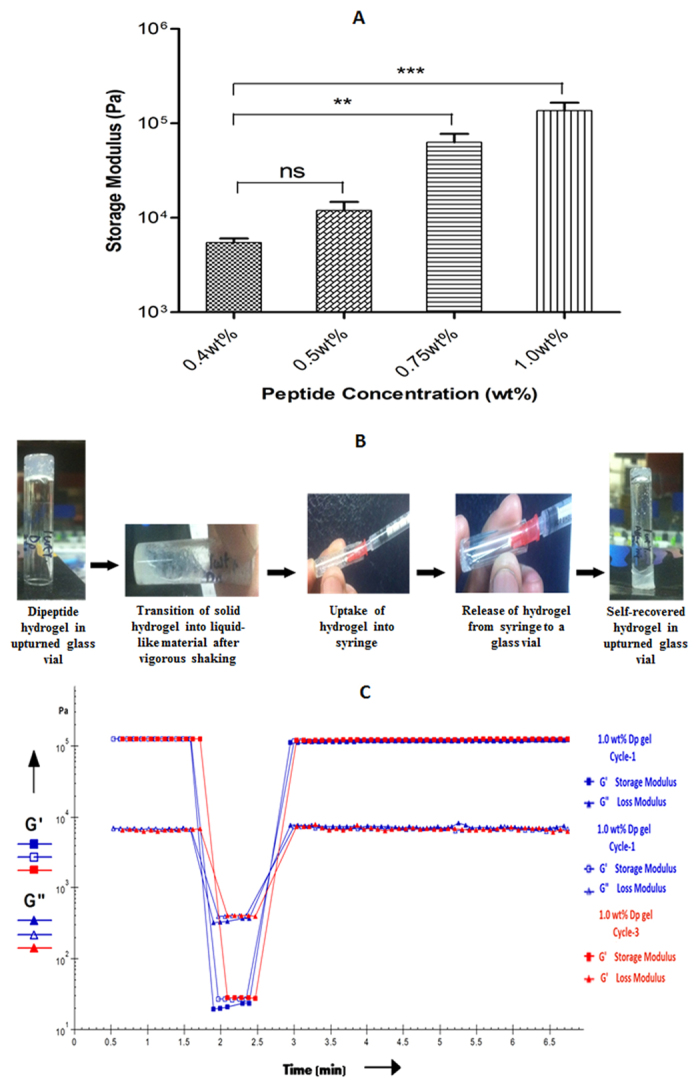
Mechanical strength and self-recovery of Dp gel **(A)** Storage modulus (G’ values) of Dp gel at different peptide concentrations (0.4wt%, 0.5 wt%, 0.75wt% and 1.0wt%), showing increase in gel strength with increasing peptide concentration Graphs represent mean ± standard deviation (n = 3). Significance values determined by t-test are marked with asterisks (ns for P-value  >  0.05, * for P-value  <  0.05, ** for P-value  <  0.01, *** for P-value  <  0.001, **** for P-value  <  0.0001). **(B)** Syringeability results showing recovery of gel (1.0wt%) after disruption of its structure. **(C)** Thixotropic time dependent step-strain data of Dp gel (1.0wt%) showing self-healing property of Dp gel. Higher G’ values and G” values (G’ > G”) initially at lower strain (0.1%) indicate solid like structure of the gel while on increasing strain to 50%, G’ and G” values were declined (G’ < G”), indicating transition of gel into liquid-like material. Finally on removal of high strain, the gel restored its original strength (G’ > G”). Three cycles were performed to verify reproducibility.

Appropriate mechanical strength, and stability, especially under physiological conditions are key factors for hydrogels to be considered as delivery platforms. To measure mechanical strength of the gel, storage modulus (G’) values were measured and we found that G’ values increased with increase in peptide concentration (5.46 ± 0.58 kPa at 0.4wt%, 12.0 ± 2.7 kPa at 0.5wt%, 63.2 ± 14.0 kPa for 0.75wt% and 136.0 ± 29.8 kPa at 1.0wt%) (Fig. 3A). Clearly gel strength increased with increasing peptide concentration. These G’ values compare extremely well with other reported peptide based hydrogels like for RADA16 gel (8 kPa)^21^, for LN-NS gel (7 kPa)^21^, for polypeptide-DNA gel (5 kPa)^22^ and for Fmoc–FF gel (10 kPa)^23^.

Frequency sweep rheology experiment (1–100 rad/s) carried out with Dp gels (0.5wt% and 1.0wt%) showed that the G’ and G” (loss modulus) values were independent on frequency suggesting their high stability (Supplementary information Figures S-5A and S-5B).

In recent years, increasing efforts have been made to design and develop hydrogels with self-healing properties for their applications in drug delivery and cell therapy purposes^24^,^25^,^26^. To explore self-healing behaviour of Dp gel, 0.5wt% and 1.0wt% Dp gels were subjected to syringeability and time dependent step-strain rheological tests^27^,^28^,^29^. Upon structural disruption and passage through the syringe needle, 0.5wt% Dp gel was not able to fully recover its strength while at 1.0wt% the gel regained its original strength as observed in tube inversion test (Fig. 3B). Time dependent step-strain rheology experiment was done on Dp gel (1.0wt%) in which a low strain (0.1%) was applied initially followed by an increase in strain to 50%, representing the force experienced by hydrogels during injection and finally reducing the strain back to 0.1%. Dp gel showed stable and higher G’ values and G” values (G’ > G”) initially representing its solid like structure, but when the applied strain was increased to 50%, the gel was disrupted and G’ and G” values were rapidly declined and become G’ < G”, representing transition of solid gel into liquid-like material. Upon removal of high stress the gel restored its original strength (G’ > G”) (Fig. 3C). Taken together results of these experiments clearly suggest that the Dp gel has the ability to withstand stress and regain its original structure.

**Figure 4:**
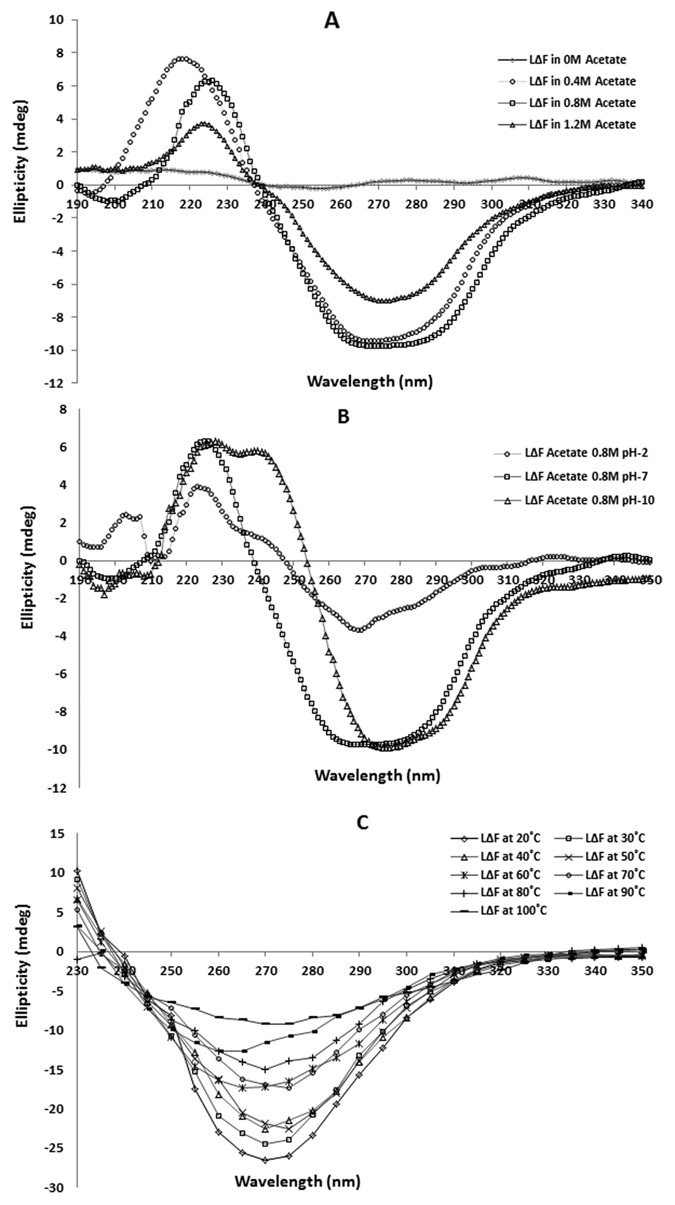
CD spectra showing effect of different parameters on formation of Dp gel and its thermal stability **(A)** CD spectrum showing conformational changes in the structure of LeuΔPhe on self-assembly in different salt (acetate) conditions. **(B)** CD spectrum showing effect of changes in pH of the buffer (sodium acetate buffer) condition on LeuΔPhe self-assembly into hydrogel. **(C)** CD temperature scan of Dp gel (0.5wt%) showing its stability at physiological temperatures.

Since peptide self-assembly is sensitive to changes in environmental conditions (like pH, salt concentration, temperature etc.), it is essential to identify and optimize suitable conditions for the development of these systems. Self-assembly of LeuΔPhe at different salt concentrations was probed by CD spectroscopy. An increase in concentration of sodium acetate saw the appearance of a negative peak at 275 nm (at 0.4 M and 0.8 M) which decreased in intensity at higher salt concentration (1.2 M) (Fig. 4A). As also reported for other hydrogels^30^,^31^; there seems to be a critical salt concentration above which self-assembled structures become unstable. Self-assembly of LeuΔPhe in different pH conditions was also investigated. Intensity of the band at 275 nm decreased by 40% at pH 2 compared to at neutral pH, and a blue shift from 275 nm at neutral to 280 nm at alkaline pH (pH 10) was observed (Fig. 4B). Tube inversion test also showed that LeuΔPhe did not form gel like structure at both acidic (pH-2) and basic pH (pH-10) (supplementary information Figure S-6). This could be due to alteration of NH_2_/NH_3_^+^ or COOH/COO^−^ content in the peptide on pH changes as also reported for other peptide based gels^21^,^32^,^33^.

Thermal scanning CD spectroscopy with Dp gel (0.5wt%) showed no significant changes in the 275 nm band till 50 °C. However at higher temperatures more than 60 °C there was significant decrease in the intensity at 275 nm, suggesting that the gel may not be stable above this temperature (Fig. 4C). Similar thermal stability patterns have also been reported for other peptide based gelators including PheΔPhe, N-terminal protected dipeptide and h9e polypeptide (19 amino acids)^13^,^34^,^35^. One of the major shortcoming of peptide based systems is their high susceptibility to enzymatic degradation under *in-vivo* conditions^36^. TEM images of Dp gel treated with trypsin and cell culture supernatant showed no observable changes in the fibrillar network compared to the untreated gel (Supplementary information Figure S-7A,S-7B). Tube inversion test of Dp gel after trypsin and cell culture supernatant treatment also showed no changes in its strength even after 5 days of incubation (Supplementary information Figure S-7C,S-7D). RP-HPLC analysis further supported these results (Supplementary information Figure S-7E), suggesting its suitability for *in vivo* & cell culture applications.

**Figure 5:**
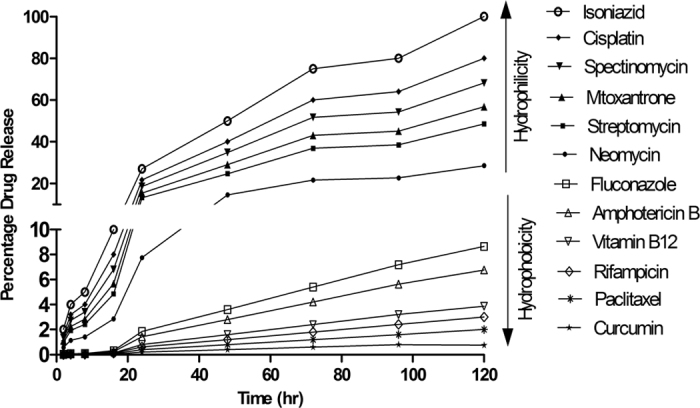
Percentage release of drugs from Dp gel, indicating their continuous & slow release.

A major factor that restricts development of hydrogels as drug delivery vehicles is their inability to effectively entrap poorly water soluble drugs due to their own extreme hydrophilic nature^37^,^38^ and, if it happens, large aggregates may form and give a burst release with high local concentration resulting in toxicity at the site of administration^38^. We tested entrapment in and release from the gel matrix of various drugs including some well known anti-cancer, anti-fungal, anti-TB and antibiotics covering a wide range of molecular weights, charge and physiochemical properties (Supplementary information Table-S2). Interestingly, both hydrophilic and hydrophobic drug molecules were efficiently entrapped in the gel. The unprotected N- and C- terminals in LeuΔPhe lend polar character while the side chains of Leu and ΔPhe provide a strong hydrophobic character and this amphipathic nature is likely responsible for the entrapment of both hydrophilic and hydrophobic drugs in the gel matrix. Entrapment of water insoluble drug, curcumin, showed a uniform distribution inside the gel with no observable aggregates (Supplementary information Figure S-8)^30^. Next, release of entrapped drugs was investigated and we found that the drug molecules, both hydrophilic as well as hydrophobic, released in a slow and continuous manner without any burst release (Fig. 5).

Diffusion coefficient values (D) of the drugs released ranged from 0.01 × 10^−10^ for a hydrophobic drug like curcumin to 6.0 × 10^−10^ for a hydrophilic drug like isoniazid. In case of hydrophilic drugs, the D-values correlated with their ClogP values, molecular weight and net charge of drugs. There was no correlation between release of hydrophobic drugs with their molecular mass, but their D-values decreased with increase in their ClogP values (Supplementary information Figure S-9A), suggesting a determining role of hydrophobic interactions between drug molecules and the gel matrix. We observed that D-values of hydrophilic drugs decreased with increase in molecular weight and net charge of drugs, and increased with increase in their clog p values (Supplementary information Figure S-9B–9D), this is likely to be due to gel matrix controlled movement of drugs and presence of electrostatic or hydrophobic interactions between drugs and gel matrix. These results suggested that the controlled release of hydrophilic drugs from Dp gel may be affected by any of the three parameters i.e. molecular mass, net charge or ClogP values of these drugs.

Conventional therapy involves frequent dosing of therapeutic drugs causing various adverse effects and poor patient compliance because of under or over dosage of drugs^39^. Sustained and controlled release formulations are likely to improve therapeutic index of drugs. The release pattern of both hydrophilic and hydrophobic drugs from the Dp gel was superior to other peptide and polymer based reported hydrogels. For example, for paclitaxel, the release from Dp gel was ~6 times slower than RADA 16 gel^40^ while mitoxantron was completely released within 8 hrs from another dipeptide hydrogel^13^. Thus, Dp gel can entrap a wide range of hydrophobic and hydrophilic drug molecules and act as a reservoir for controlled release of drugs.

In order to achieve a stimuli responsive release of drugs, it is necessary for the delivery system to be able to sense and respond to small changes in the external environment like pH, and temperature^41^ To investigate if the release of an entrapped drug can be modulated by changes in pH, we chose curcumin as an example. When the pH was changed from neutral to acidic (pH-3 and pH-5.6) or basic pH (pH-8.5 and pH-10), the cumulative percentage release of curcumin from Dp gel (0.5wt%) was increased substantially (Supplementary information Figure S-10). Similar pH responsive drug delivery from peptide based hydrogels has been also reported in literature^32^,^42^,^43^.

**Figure 6:**
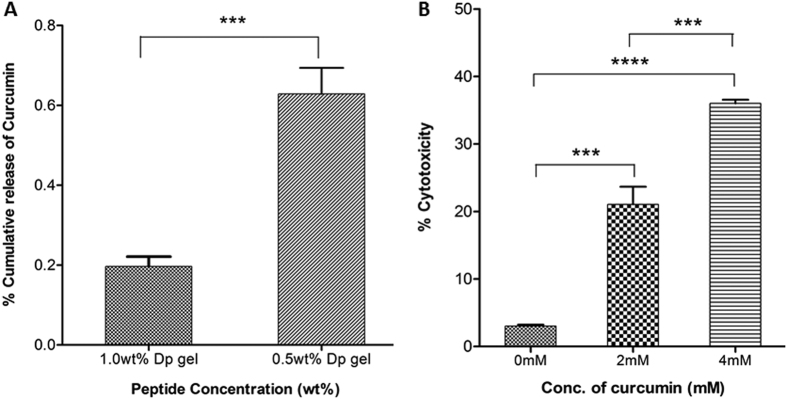
(**A**) Cumulative percentage release of curcumin from Dp gels (at 0.5wt% and 1wt%) showing decrease in curcumin release on increase in peptide concentration, indicating its peptide concentration based tunability. **(B)** Percentage cytotoxicity of released curcumin on HeLa cells from Dp gels (0.5wt%), entrapped with 0 mM, 2 mM and 4 mM curcumin, assessed by LDH assay. Graphs represent mean ± standard deviation (n = 3). Significance values determined by t-test are marked with asterisks (ns for P-value  >  0.05, * for P-value  <  0.05, ** for P-value  <  0.01, *** for P-value  <  0.001, **** for P-value  <  0.0001).

In peptide based hydrogels, concentration of the peptide can also play a key role in regulating their mesh size and other interactions responsible for holding drugs inside the gel matrix^44^. Also, for any drug delivery system, retention of bioactivity of entrapped drugs is essential for their potential applications in clinic. To address these issues, we again chose curcumin^45^ and found that the amount of curcumin released over time (48 hr) decreased with increasing peptide concentration from 0.5wt% to 1.0wt% (Fig. 6A), this is likely due to increased fibrilar mesh and hydrophobic interactions between drug molecules and fibrilar network of the gel, as has been reported for other hydrogels^46^,^47^. To determine the activity of drug entrapped in the gel matrix, Dp gels (0.5wt%) entrapped with different concentrations of curcumin were exposed to HeLa cells and cytotoxicity of the curcumin released was monitored by LDH assay. Significantly higher cell death was observed at 4 mM (35.6 ± 0.53%) than at 2 mM (22 ± 2.61%) curcumin entrapped gel while Dp gel alone did not show any cytotoxicity (Fig. 6B). These results suggested that not only the amount of drug release may be regulated by changing the peptide concentration or amount of drug entrapped in the peptide gel but also that a drug like curcumin retains its activity.

**Figure 7:**
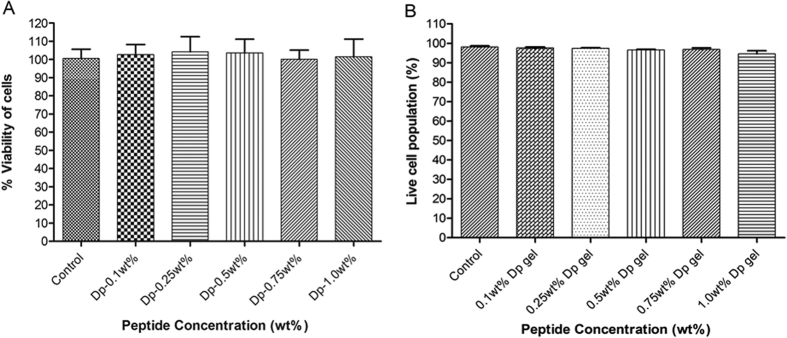
Cytotoxicity of Dp gel **(A)** Percentage cell viability of HEK293T cells treated with Dp gels of different peptide concentration, indicating its biocompatibility. **(B)** Live/Dead assay results showing no change in percentage population of live cells in the presence of Dp gel compared to control, indicating its high biocompatibility. Graph represents mean ± standard deviation (n = 3).

In the development of new materials for drug delivery and other biomedical applications, biocompatibility is the prerequisites criterion for their clinical use^48^,^49^. Biocompatibility of Dp gel was assessed by MTT and live/dead assay using HEK293T cells. In MTT and live/dead assay, HEK293T cells were exposed to gels of different peptide concentrations and we found that the growth of cells was unaffected even at higher peptide concentrations (Fig. 7A,B) (Supplementary information Figure S-11), indicating no cellular cytotoxicity and high biocompatibility of the gel.

**Figure 8:**
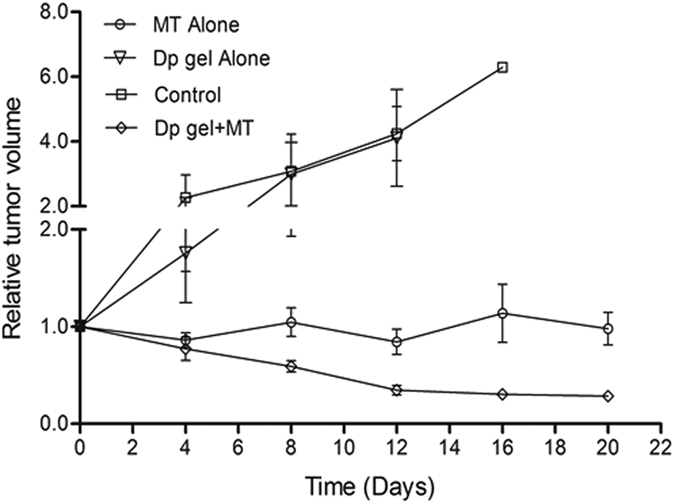
Tumor regression study in mice xenograft model showing changes in relative tumor volume of different groups of mice treated with different formulations with time. Graph represents mean ± standard deviation (n = 4).

We next investigated whether Dp gel can be useful for *in-vivo* delivery purposes. For this as a proof of principle, we used a mice xenograft tumor model and mitoxantrone as the anticancer drug. Four groups of tumor bearing mice were treated with PBS, Dp gel, mitoxantrone (MT) and MT entrapped in Dp gel. In mice receiving PBS or Dp gel alone, a continuous tumor growth was observed. In mice treated with MT alone, while growth was arrested there was no reduction in the size of tumors (Fig. 8), suggesting that MT was more effective when delivered as entrapped in the gel. There was no body weight loss in mice injected with Dp gel alone, suggesting its biocompatibility *in vivo*. A minor decrease in body weight was observed in mice treated with MT and MT entrapped in Dp gel (Supplementary information Figure S-12). During cancer treatment, chemotherapy involves various side effects due to toxicity of anticancer drugs and may cause body weight loss. Such weight loss in mice treated with anticancer drugs have also been reported earlier^50^,^51^. All these results suggest that LeuΔPhe forms a biocompatible hydrogel suitable for *in-vivo* applications.

## Methods and Materials

### Synthesis of Leucine-α,β-dehydrophenylalanine

Dipeptide LeuΔPhe was synthesized at 5 mM scale by solution phase peptide synthesis described earlier (Supplementary Information text)^8^,^9^.

### Preparation of dipeptide hydrogel

LeuΔPhe was dissolved in minimum amount of methanol (50 mg/mL) using 10 min sonication. Instantaneous dipeptide gel formation was achieved by addition of 0.8 M sodium acetate buffer pH 7 to the peptide solution at room temperature.

### Turbidity

Peptide concentration dependent self-assembly of LeuΔPhe was investigated by monitoring turbidity of Dp gel prepared at different concentrations (0.05wt%, 0.1wt%, 0.2wt%, 0.3wt%, 0.4wt%, 0.5wt%, 0.75wt% and 1.0wt%) using U.V spectroscopy at 360 nm (Supplementary information text)^11^,^12^.

### Dynamic light scattering (DLS) studies

Self-assembly kinetics of LeuΔPhe at different concentrations (0.05–0.4 wt%) was investigated by DLS. DLS studies at different time points (1 hr, 2 hr, 3 hr, 4 hr, 5 hr and 6 hr) were probed using Zetasizer Nano-ZS (Malvern Instruments) at a light scattering angle of 90° using 632 nm laser. All studies were conducted at room temperature.

### Thioflavin-T (ThioT) fluorescence assay

Self-assembly kinetics of LeuΔPhe at different (0.05–0.4 wt%) peptide concentrations was again determined by ThioT fluorescence assay using Victor fluorescence multi-well plate reader (PerkinElmer) at 440 nm excitation and 482 nm emission filter. Freshly prepared ThioT dye was added (2 μl of 2 mM stock per 200 μl sample) to the samples at different time intervals (1 hr, 2 hr, 3 hr, 4 hr, 5 hr, 6 hr, 7 hr and 8 hrs) and fluorescence was measured using fluorescence spectrophotometer.

### Transmission electron microscopy (TEM)

Samples for TEM analysis were prepared by negative staining method. A 10 μl of each gel sample (diluted to 1:1 ratio with water just before sample loading) was spotted on a 200 mesh 3 mm carbon coated nickel grid, stained with 1% uranyl acetate and viewed under the 120 kV mode of a transmission electron microscope (Tecnai 12 BioTWIN, FEI Netherlands). Photomicrographs were digitally recorded using a Megaview II (SIS, Germany) digital camera. Image analysis was carried using Analysis II (Megaview, SIS, Germany) software packages.

### Scanning Electron Microscopy (SEM)

A 0.5 wt% gel sample was spreaded over the double sided tape already placed on the SEM holder and gold coated by ion sputtering (E1010, Hitachi, Japan) for 30 s at operating vacuum of 10 Pa. SEM images were acquired with a ZEISS EVO 50 (Carl Zeiss Microscopy, GmbH, Germany) with 2.0 nm resolution at an acceleration voltage of 0.2 to 30 kV.

### Environmental scanning electron microscopy (ESEM)

Environmental scanning electron microscopy (ESEM) images were recorded using a field emission scanning electron microscope (FE-SEM), Quanta 200F, FEI (Eindhoven, Netherlands). Dp gel (0.5wt%) was spreaded over carbon tape placed on specimen holder an imaged. Images were obtained in environmental wet mode at a low vacuum and collected using large-field gaseous secondary electron detector (GSED).

### Circular dichroism (CD) spectroscopy

CD experiments were carried out in a 0.1 cm path length quartz cuvette using JASCO-810 polarimeter equipped with peltier temperature controller at 0.2 mg/ml peptide concentration. UV-CD (from 190 nm to 350 nm) spectra were taken for gels prepared in buffers having different sodium acetate concentrations (0 M–1.2 M) to investigate the effect of salt concentration on gelation. To monitor the effect of pH on self-assembly of LeuΔPhe, the CD spectra of gels prepared at different pH conditions i.e. acidic and basic, and neutral were collected. To investigate the thermal stability of gel, CD spectra were collected in the temperature range of 20–100 °C. An average of 5 scans was used for all spectra.

### Fourier Transform Infrared (FTIR) Spectroscopy

IR spectra of 0.5wt% Dp gel were collected using Varian FTIR spectrometer to determine molecular structure of Dp gel (Supplementary Information text).

### Syringeability and rheology Studies

Syringeability and rheology experiments were performed to determine strength, stability and self-healing property of the Dp gel (Supplementary Information text)^27^,^28^,^29^.

### Drug loading & release

Drug loaded Dp gels (0.5wt%) were prepared by incorporating drugs (to a final concentration of 4 mM) during gel formation and were kept undisturbed for 30 min, washed three times with PBS to remove the unentrapped drug. For release study, 5 mL of PBS was placed on top of the gels and 1.0 mL of samples were withdrawn from the release medium at different time intervals (2 hr, 4 hr, 8 hr, 16 hr, 24 hr, 48 hr, 72 hr, 96 hr and 120 hr) and replaced with fresh PBS. Contents of both hydrophilic and hydrophobic drugs into the overlying PBS was monitored by methods reported earlier^13^,^52^,^53^,^54^,^55^,^56^ using UV-vis spectroscopy. The percentage release was calculated using an equation: Percentage Release = (Absorbance of drug released in PBS/Absorbance of total drug encapsulated in gel) X 100. To study the effect of environmental pH on release rate of drugs entrapped in the peptide hydrogel, Dp gels (0.5wt%) were prepared by incorporating curcumin (4 mM), kept undisturbed for 30 min and washed three times with PBS to remove the unentrapped drug. The pH of the media was adjusted to pH-3, 5.6, 7.4, 8.5 and 10 using 0.1 M HCl and 1 N NaOH and 5 ml of the respective media was placed on top of the gels. 1.0 ml samples were withdrawn from the release media at different time intervals (1 hr, 2 hr, 4 hr, 8 hr, 24 hr & 48 hr) and replaced with fresh 1 ml media. The amount of curcumin released was determined by UV-vis spectroscopy^13^,^56^.

The relative diffusion coefficient (D) of drugs in the gel matrix was calculated by using the non-steady state diffusion model equation given as: M_t_/M_∞_ = 4 X (D_t_/π λ^2^)^1/2^, where, M_t_ is the total amount of drug released at the time of measurement, M_∞_ is the total amount of drug that was kept in the matrix, λ represents the hydrogel thickness, t is the time of measurement, and D is the diffusion coefficient of the drug^55^. The ClogP values of the drugs were obtained from “drug bank” and “pubchem”.

### Activity of drug entrapped in Dp gel

Dp gels (0.5wt%) containing different curcumin concentrations (0 mM, 2 mM and 4 mM) were prepared, washed with PBS and transferred to 24-well 0.4 μm pore size polyester transwells. HeLa cells were plated (1 × 10^5^ Cells/well) in TCTP 24-well plate and incubated overnight. Next day, transwells containing curcumin entrapped gels were placed into 24-well plate containing HeLa cells and incubated for 3 hrs at 37 °C. Cells were imaged for morphological changes due released curcumin and cytotoxicity was determined using standard LDH assay^57^. All experiments were performed in triplicate.

### Cell viability and live/dead assay

Hek293T cells were grown in DMEM culture media supplemented with 10% FBS and 0.1% gentamycin respectively, on TCTP culture plates at 37 °C in a humidified atmosphere containing 5% CO_2_. Subconfluent cells were trypsinized, harvested and counted using hemocytometer. Cell suspensions were diluted with culture medium and seeded at a concentration of 1 × 10^3^ cells/well in TCTP 96-well plate for MTT (methyl thiazolyl tetrazolium) assay and 5 × 10^4^ cells/well in 24 well plate for live/dead assay. The plates were incubated at 37 °C in a humidified atmosphere containing 5% CO_2_ for 24 hr to allow cell spreading and attachment. Dp gels of different peptide concentrations (0.1wt%, 0.25wt%, 0.5wt%, 0.75wt% & 1.0wt% gel) were prepared and kept in hood overnight for methanol evaporation. Next day, gels (50 μl each in triplicate) were added to the respective wells and incubated for 24 hr. Cells treated with culture medium alone were used as control group. Cell viability was determined by standard MTT assay^57^ while live/dead assay was performed using FACS^58^.

For live/dead assay, after 24 hr treatment, cell culture supernatants were removed and cells were trypsinized (100 μl/well), neutralized with DMEM culture media (1 ml/well) and transferred to eppendorf tubes. Cells were washed and resuspended in FACS buffer (PBS containing 0.2% FBS) before staining. eFluor^®^ 780 staining solution was prepared freshly in FACS buffer, added (50 μl each) to the cell suspensions and incubated for 30 min in dark. Unbound dye was removed by washing with FACS buffer (2 times) and samples were processed on FACS CANTO-II flow cytometer (BD Bioscience, San Jose, CA, USA). Fluorescence of eFluor^®^ 780 was collected in the APC-Cy7 channel, equipped with a 780 nm band pass filter. A total of 1 lakh events from each sample were acquired to ensure adequate data, and histograms representing live/dead population of cells were plotted using FlowJo software (FlowJo Enterprises).

### *In vivo* efficacy of Dp gel

To determine *in vivo* efficacy of Dp gel as drug reservoir for controlled release of drugs, tumor regression study using mice xenograft model was carried out^59^. Dp gel containing mitoxantrone (4 mM) was prepared and incubated in laminar hood overnight in dark for methanol evaporation. Unentrapped drug was separated from entrapped drug by centrifugation (800 x g for 15 minutes) using 4 ml Amicon Ultra-4 centrifugal filter (Ultracel – 3 kDa, Merck Millipore Ltd. Tullagreen, Carrigtwohill). Female mice (Balb/c), 4–6 weeks old, weighing 18–22 g were housed in a temperature and light controlled room. All animal experiments were approved by the Institutional Animal Care and Use Committee at International Centre for Genetic Engineering & Biotechnology, Delhi, India and were in compliance with all regulatory guidelines. B_16_F_10_ Cells (Mouse melanoma cells) were used and cultured in RPMI 640 culture media supplemented with 10% FBS and 0.1% gentamycin for tumor generation in mice. Subconfluent B_16_F_10_ cells were trypsinized, harvested, washed thrice with cold PBS and counted using hemocytometer. Cell suspension was again diluted with cold PBS to a concentration of 1 × 10^6^ cells/100 μl of PBS. Each mouse was injected subcutaneously with 1 × 10^6^ B_16_F_10_ cells in the abdomen for tumor generation. After 14 days of tumor cell inoculation, mice were divided into four different groups (4 mice each group) i.e. control (treated with PBS), Dp gel alone, mitoxantrone alone and Dp gel entrapped with mitoxantrone. Tumor bearing mice were treated with different gel samples (intraperitoneal injection near tumor) after every 7 days (0^th^, 8^th^ and 16^th^ day). Every 4^th^ day, changes in tumor size were monitored using vernier calliper and the day of first injection was taken as 0^th^ day. Mice weight was also recorded and tumor volume and relative tumor volume were calculated using equations^60^: Tumor Volume = (L X W^2^)/2 and relative tumor volume = (TV_n_)/(TV_o_), where, L and W are length (longest diameter) and width (shortest diameter) of tumor (in mm) respectively and TV_o_ and TV_n_ are the volume of tumor (in mm^3^) on 0^th^ and n^th^ day respectively.

### Proteolytic Stability of the Dipeptide Gel

Enzymatic stability of Dp gel was determined by incubating Dp gel with cell culture supernatant and trypsin (a serine protease) solution for 5 days. To examine gel stability, RPHPLC, tube inversion test and TEM imaging were carried out.

### Statistical Analysis

All statistical analyses were carried out using SigmaStat 5.0 (Systat Software, San Jose, Ca) and data presented as mean values ± SD.

## Conclusion

We have found that an ultrashort peptide containing α, β-dehydrophenylalanine, LeuΔPhe, spontaneously forms strong and stable hydrogel under physiological conditions. LeuΔPhe forms hydrogel at relatively low concentrations and has high mechanical strength and stability to shear stress under physiological conditions. The gel efficiently entrapped a number of hydrophobic and hydrophilic drug molecules and released them in a controlled manner. The gel entrapped and released mitoxantrone and significantly controlled tumor growth in an *in-vivo* mouse model. Its features like ease of synthesis and derivatization, high mechanical strength, injectability, proteolytic stability, non-cytotoxicity, ability to entrap and release drug like molecules make it an attractive candidate for drug delivery.

## Additional Information

**How to cite this article**: Thota, C. K. *et al*. “A novel highly stable and injectable hydrogel based on a conformationally restricted ultrashort peptide”. *Sci. Rep*. **6**, 31167; doi: 10.1038/srep31167 (2016).

## Supplementary Material

Supplementary Information
