# Supplementary material for: “A novel highly stable and injectable hydrogel based on a conformationally restricted ultrashort peptide”
Source: Sci Rep. 2016 Aug 10;6:31167. doi: 10.1038/srep31167 (PMC4979021; doi:10.1038/srep31167)
Supplement: Supplementary Information [file srep31167-s1.pdf]

**Electronic Supplementary Information**

# "A novel highly stable and injectable hydrogel based on a conformationally restricted ultrashort peptide"

Chaitanya Kumar Thota [†,1], Nitin Yadav [†,1], Virander Singh Chauhan [1,*]

International Centre for Genetic Engineering & Biotechnology
Aruna Asaf Ali Marg, New Delhi-110067, India
Tel: +91-11-26742317; Fax: +91-11-26742316; E-mail: virander@icgeb.res.in

## 1. Supplementary Text

**Materials used:** Tetrahydrofuran (THF), Trifluoroacetic acid (TFA), Dimethyl sulfoxide (DMSO), Dichloromethane (DCM), Methanol, N-methyl morpholine (NMM), Isobutyl chloroformate, DL-threo-β-phenylserine, acetic acid, diethyl ether, Neomycin, Streptomycin, Mitoxanthrone, Ampicilin, Isoniazid, Amphotericin B, Qunine, Paclitaxel, Curcumin and Vitamin $B_{12}$ were purchased from Sigma, Boc-Leu-OH amino acid was purchased from Novabiochem. Sodium acetate, ethyl acetate, diethyl ether, acetic acid from spectrochem; anhydrous sodium sulfate, sodium acetate and citric acid from Merck, Dulbecco's Modified Eagle's Medium (DMEM) and Roswell Park Memorial Institute (RPMI)1640 Medium from Invitrogen; Rifampicin (Calbiochem); bromophenol blue (Bio-Rad). HeLa, Hek293T and $B_{16}F_{10}$ cells from ATCC, Tissue culture treated culture and 96-well and 24 well plates (TCTP), 12mm Transwells were purchased from Corning.

**Synthesis of Leucine-α,β-dehydrophenylalanine:** Dipeptide LeuΔPhe was synthesized by solution phase peptide synthesis. Briefly, Boc-Leu-OH (1.16 g, 5 mM) was dissolved in dry tetrahydrofuran and the resulting solution stirred in an ice-salt bath at -20°C. N-Methylmorpholine (0.5 mL, 5 mM) was added to the solution followed by isobutyl chloroformate (0.7 mL, 5 mM). After 10 min, a pre-cooled aqueous solution of DL- threo-β-phenylserine (1 g, 5.5 mM) and sodium hydroxide (0.22 g, 5.5 mM) was added and the resulting mixture stirred overnight at room temperature followed by the hydrolysis of the resultant azalactone to provide Boc-LeuΔPhe-OH. Deprotection of the Boc-LeuΔPhe-OH was achieved by treating it with a mixture of anhydrous TFA and DCM (1:1 v/v) for 30 min on ice. Excess TFA was subsequently evaporated and the compound precipitated with anhydrous diethyl ether. The precipitated compound was washed three times with diethyl ether. The resulting compound was dried, lyophilized, purified and confirmed by analytical

RPHPLC and mass spectroscopy (Supplementary information Figure S-12). Yield: 1.22 g, 88%; *Rf* was 0.36 ($CHCl_3$-MeOH, 9:1).

**Turbidity:** Dp gels (0.1wt%, 0.2wt%, 0.3wt%, 0.4wt%, 0.5wt%, 0.75wt% and 1.0wt% gels) were prepared in a U.V transparent 96 well plate and absorbance was recorded at 360nm using SpectraMax M3 Microplate Reader (Molecular devices).

**Fourier Transform Infrared (FTIR) Spectroscopy:** IR spectra were collected on a Varian FTIR spectrometer. A 0.5 wt % dipeptide gel was prepared and lyophilized into a dry powder. FTIR spectrum of LeuΔPhe gel powder was obtained using KBr pellet. Each spectrum was an average of 256 collections at a resolution of 4 $cm^{-1}$.

**Syringeability and rheology Study:** Syringeability test was done on Dp gels (05wt% and 1.00wt%) by first disrupting gels using high vertexing and then passed through a syringe with 18 gauze needle. After this, the gels were kept undisturbed to check their self-healing property. Rheology experiments were performed to determine strength, stability and thixotropic behaviour of Dp gel on Physica MCR 301 rheometer (Anton Paar) using a 25mm-diameter parallel plate geometry tool. Dp gels were prepared and immediately transferred to the rheometer bottom plate. An amplitude sweep (0.01% - 100% strain) experiment was performed at a constant oscillatory frequency of 1Hz and values of storage modulus (G') and loss modulus (G") were recorded for 0.4wt%, 0.5wt%, 0.75wt% and 1.0wt% Dp gel to determine their strength and linear viscoelastic regime (LVR). A frequency sweep experiment was also carried out for 0.5wt% and 1wt% Dp gel, where the G' and G" values were recorded as a function of angular frequency (0.1-100rad/s) at constant strain of 0.1%.

To demonstrate thixotropic behaviour of Dp gel, a time dependent step-strain rheological experiment was carried out.[27-29] In this, 1.0wt% gel was first kept under low constant strain of 0.1% and then quickly subjected to a higher strain of 50% to completely disrupt the gel

structure. Next the strain value was again reduced to 0.1% to investigate time dependent recovery of gel strength. Three cycles were performed to verify reproducibility.

## 2. Supplementary Figures

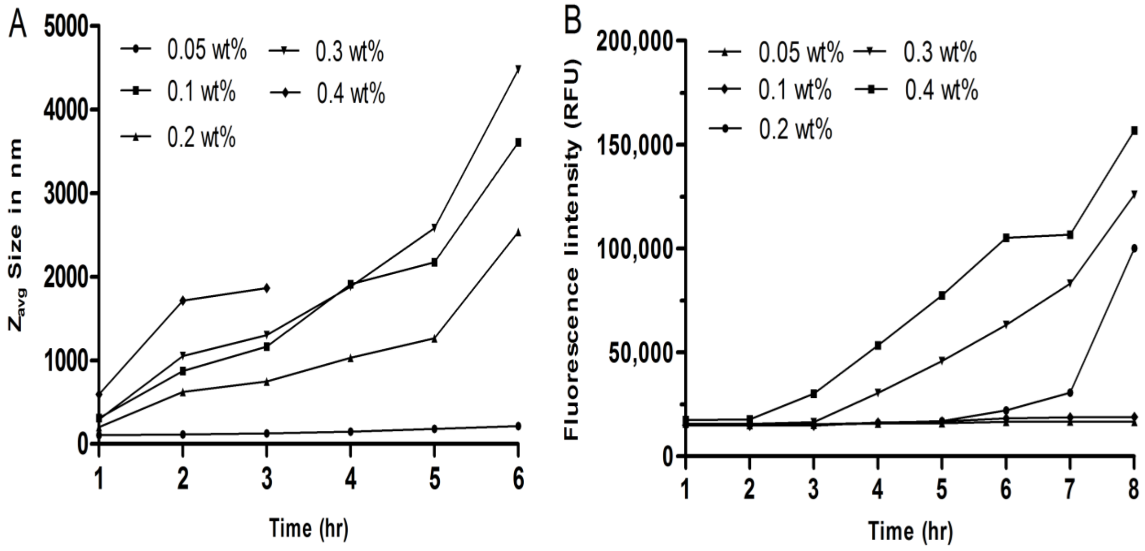


**Figure S-1.** Peptide concentration based changes in size of self-assembled peptide nanostructures **(A)** $Z_{average}$ values of self-assembled LeuΔPhe, showing increase in size of self-assembled peptide nanostructures with concentration and time. **(B)** Thiolavin T fluorescence assay showing increase in fluorescence intensity with increase in peptide concentration and time, indicating peptide concentration and time dependent self-assembly of LeuΔPhe.

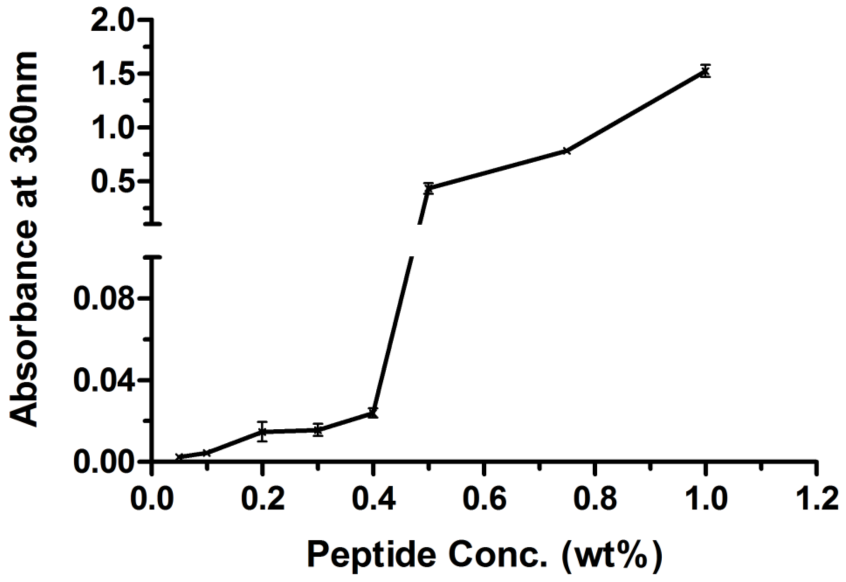


**Figure S-2.** Peptide concentration dependent self-assembly of LeuΔPhe. Graph showing changes in turbidity, OD at 360nm, of Dp gels prepared at different LeuΔPhe concentration (0.05wt%, 0.1wt%, 0.2wt%, 0.3wt%, 0.4wt%, 0.5wt%, 0.75wt% and 1.0wt%). Graph represents mean ± standard deviation (n=3).

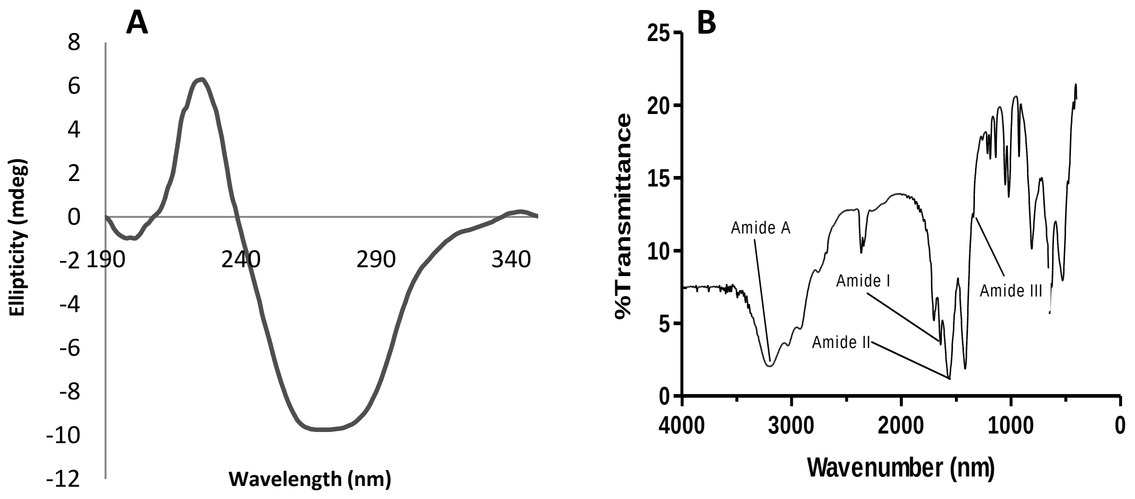


**Figure S-3. (A)** CD spectrum of Dp gel (0.5wt%), **(B)** FTIR spectra of dried powder of Dp gel (0.5wt%).

No gelation | Gelation into a strong hydrogel | No gelation

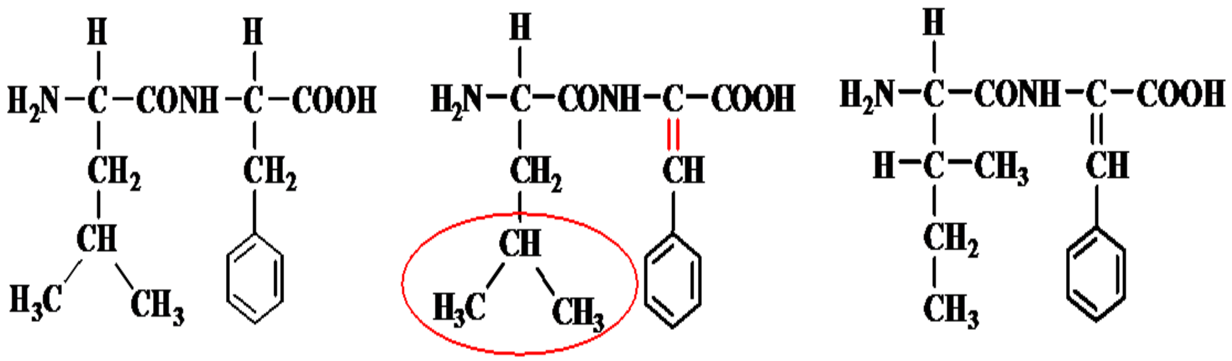

Leucine-phenylalanine | Leucine-α, β-dehydrophenylalanine | Isoleucine-α, β-dehydrophenylalanine

**Figure S-4** Chemical structures of Leucine-phenylalanine (Left), Leucine-α, β-dehydrophenylalanine (Middle) and Isoleucine-α, β-dehydrophenylalanine (Right), showing changes in nature of self-assembly of peptides due to small structural changes.

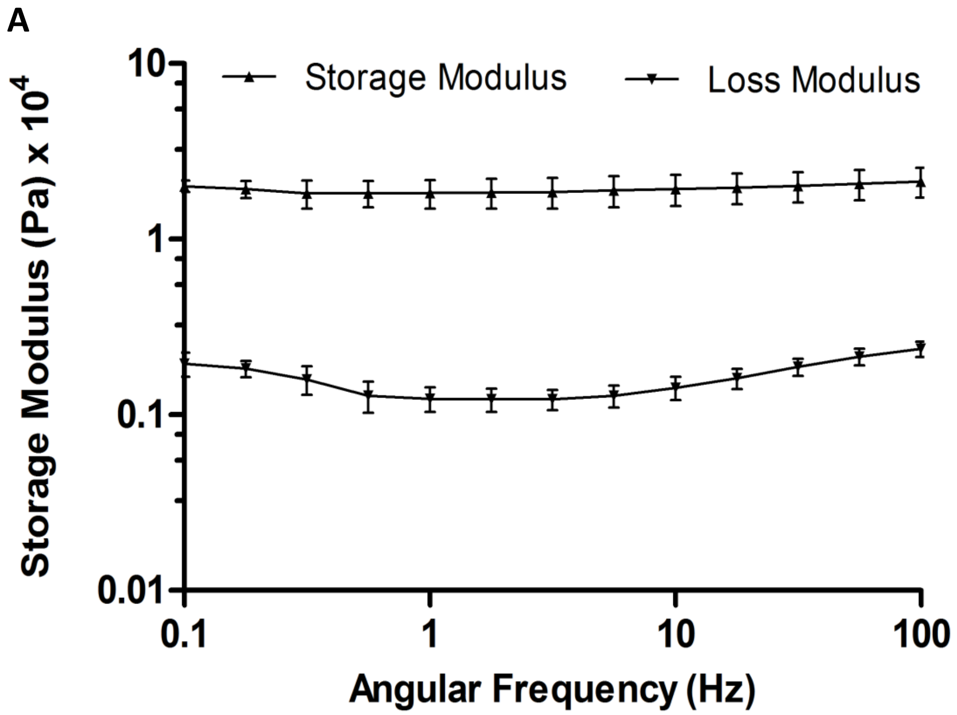

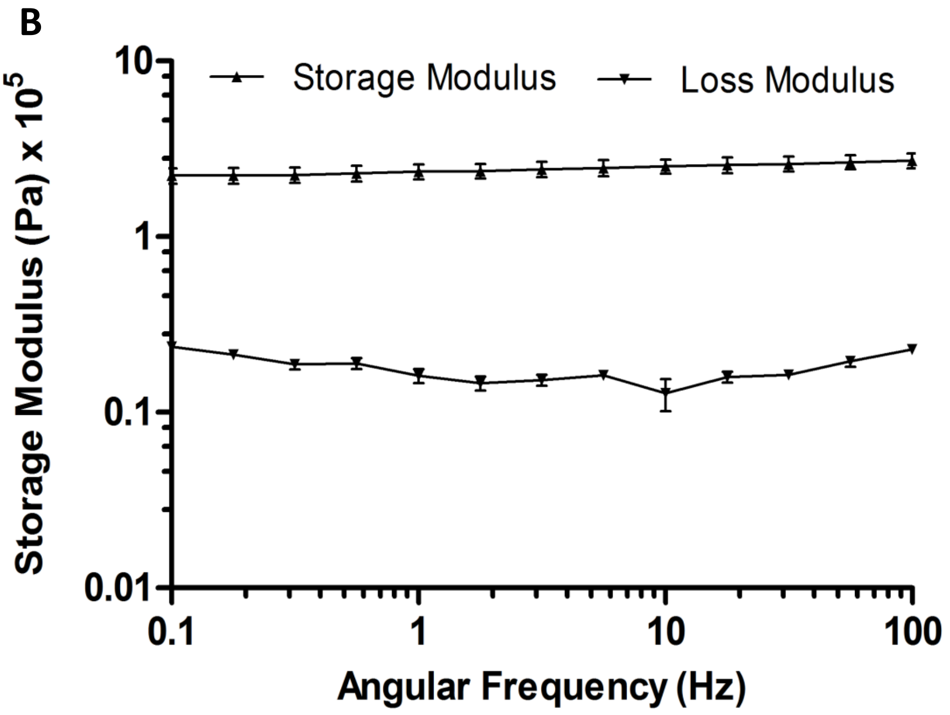


**Figure S-5.** Frequency sweep analysis (0.1-100rad/s) of **(A)** Dp gel (at 0.5wt%) and **(B)** Dp gel (at 1.0wt%), showing frequency independent storage and los modulus indicating its high stability. Graph represents mean ± standard deviation (n=3).

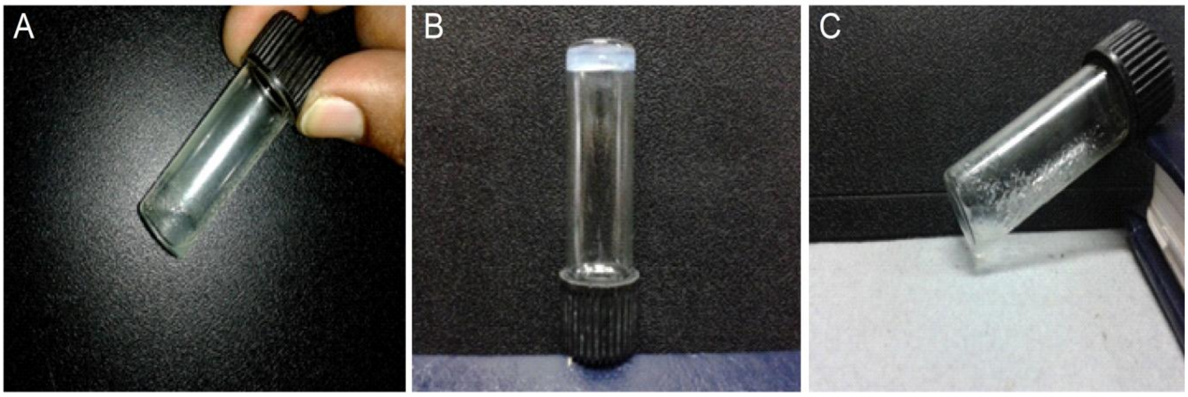


**Figure S-6.** Self assembly of Leu∆Phe at different pH conditions **(A)** In acidic condition (pH 2), showing no gelation at lower pH; **(B)** In neutral condition (pH 7) leading to gel formation; **(C)** In basic condition (pH 10), indicating no gelation at higher pH.

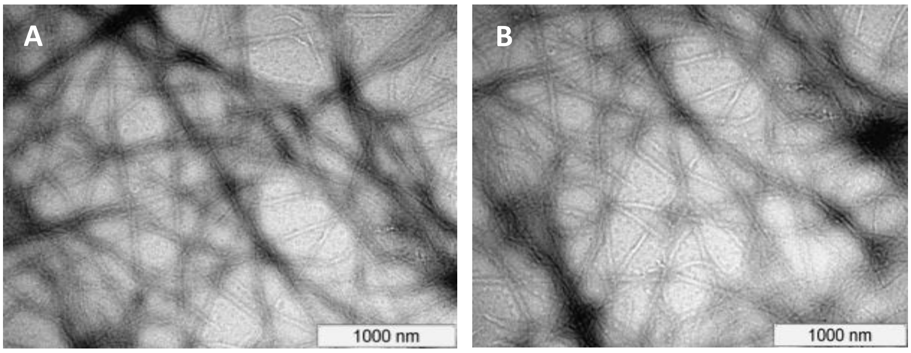


Day-1
C

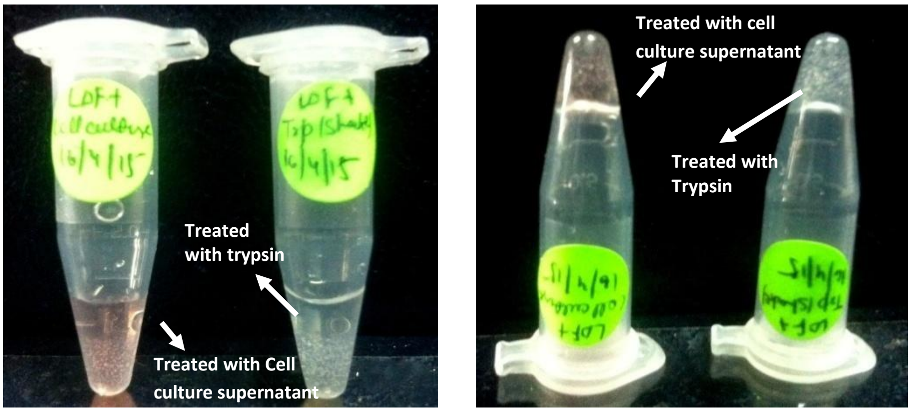


Day-5
D

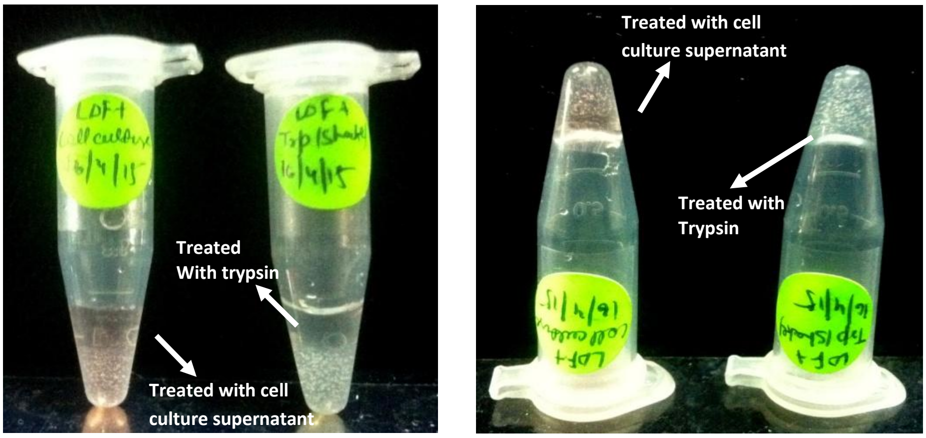


E

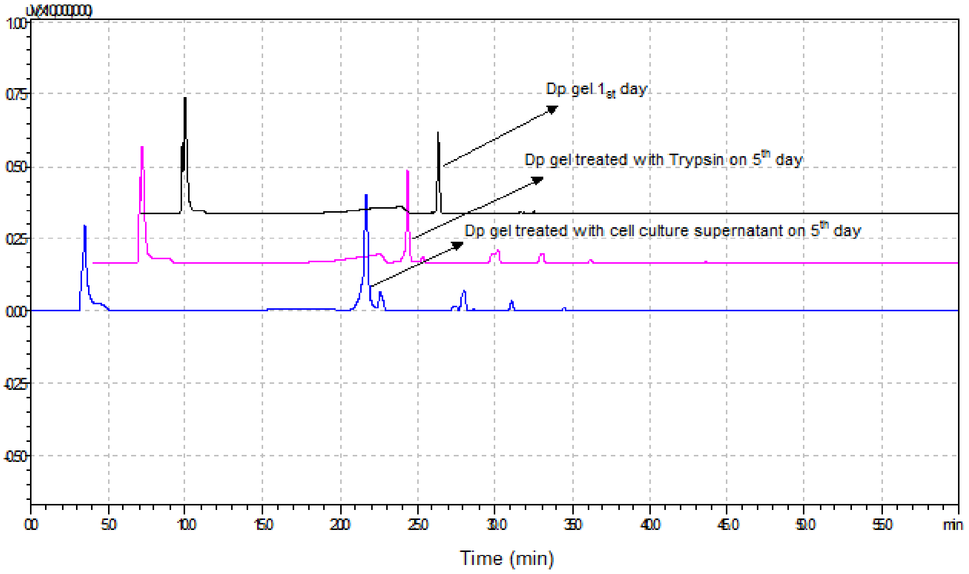

**Figure S-7. (A)** TEM images of 0.5wt% Dp gel treated with trypsin, **(B)** TEM images of 0.5wt% Dp gel treated with cell culture supernatant; showing no change in the fibrilar network of the gel. **(C)** Tube inversion test of 0.5wt% Dp gel treated with trypsin and cell culture supernatant on day-1. **(D)** Tube inversion test of 0.5wt% Dp gel treated with trypsin and cell culture supernatant on day-5 showing no change in its strength. **(E)** RP-HPLC chromatogram showing retention of LeuΔPhe peak after treatment with proteases indicating stability of Dp gel against enzymatic proteolysis..

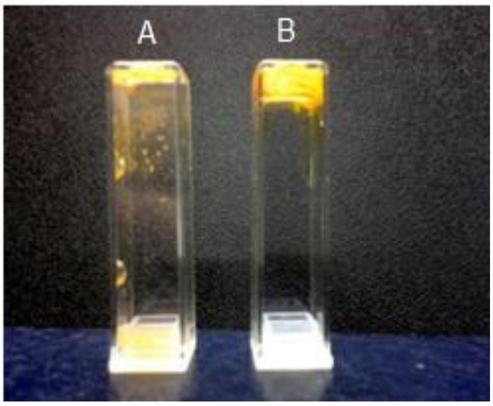


**Figure S-8. (A)** Curcumin (4mM) in 0.8M sodium acetate buffer without peptide in an upturned vial; **(B)** Dp gel (0.5wt%) entrapped with 4 mM curcumin in an upturned vial.

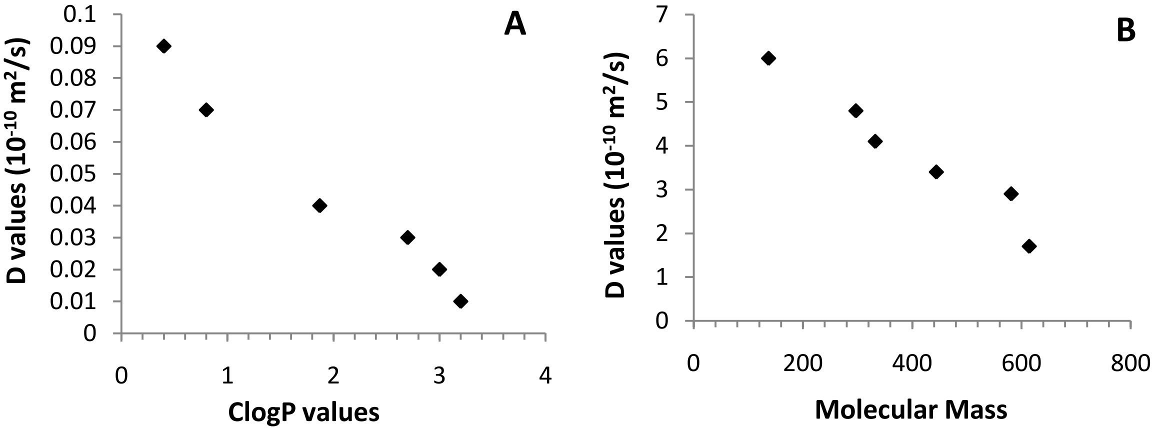

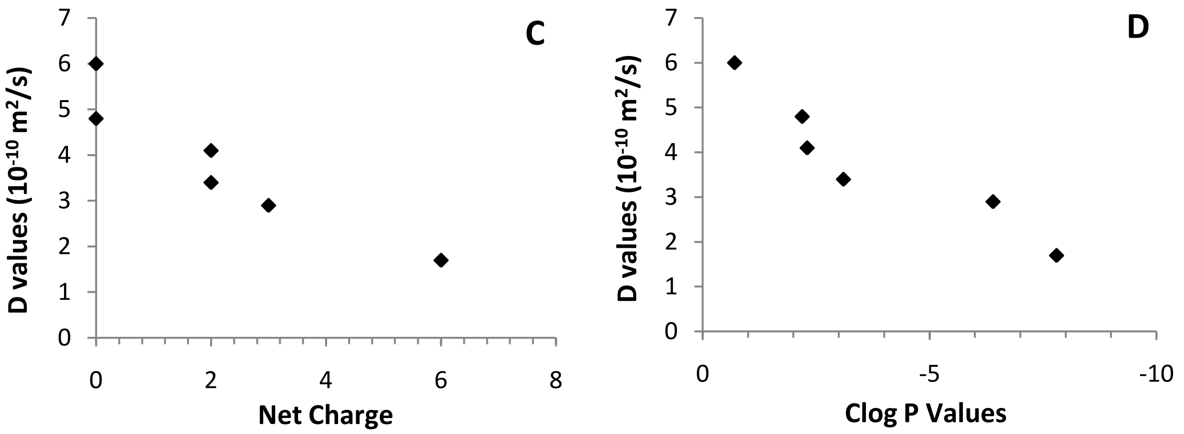


**Figure S-9.** Correlation of D values with different parameters of drug **(A)** Correlation of *D values* with ClogP values of hydrophobic drugs, **(B)** Correlation of *D values* with molecular weight of hydrophilic drugs, **(C)** Correlation of *D values* with net charge of hydrophilic drugs, **(D)** Correlation of *D* values with ClogP values of hydrophilic drugs.

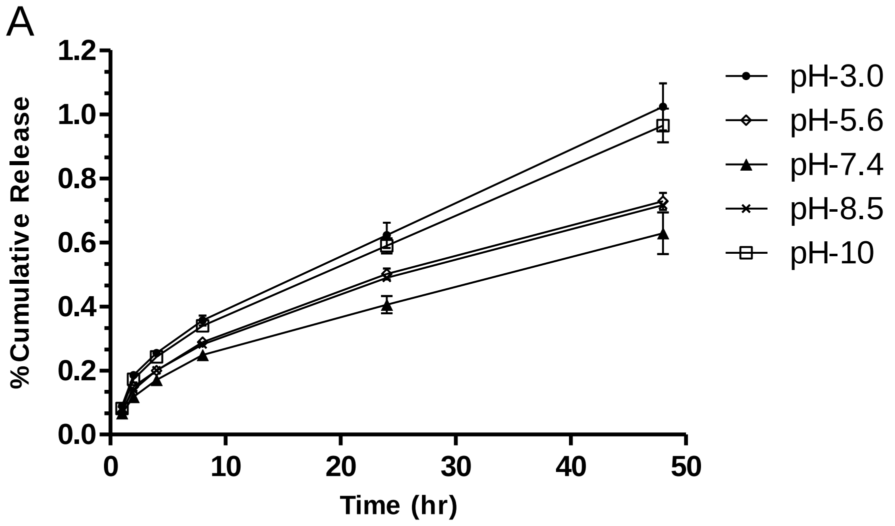


**Figure S-10.** Cumulative percentage release of curcumin at different pH conditions from Dp gel (0.5 wt%), representing pH sensitive drug release from the hydrogel. Graph represents mean ± standard deviation (n=3).

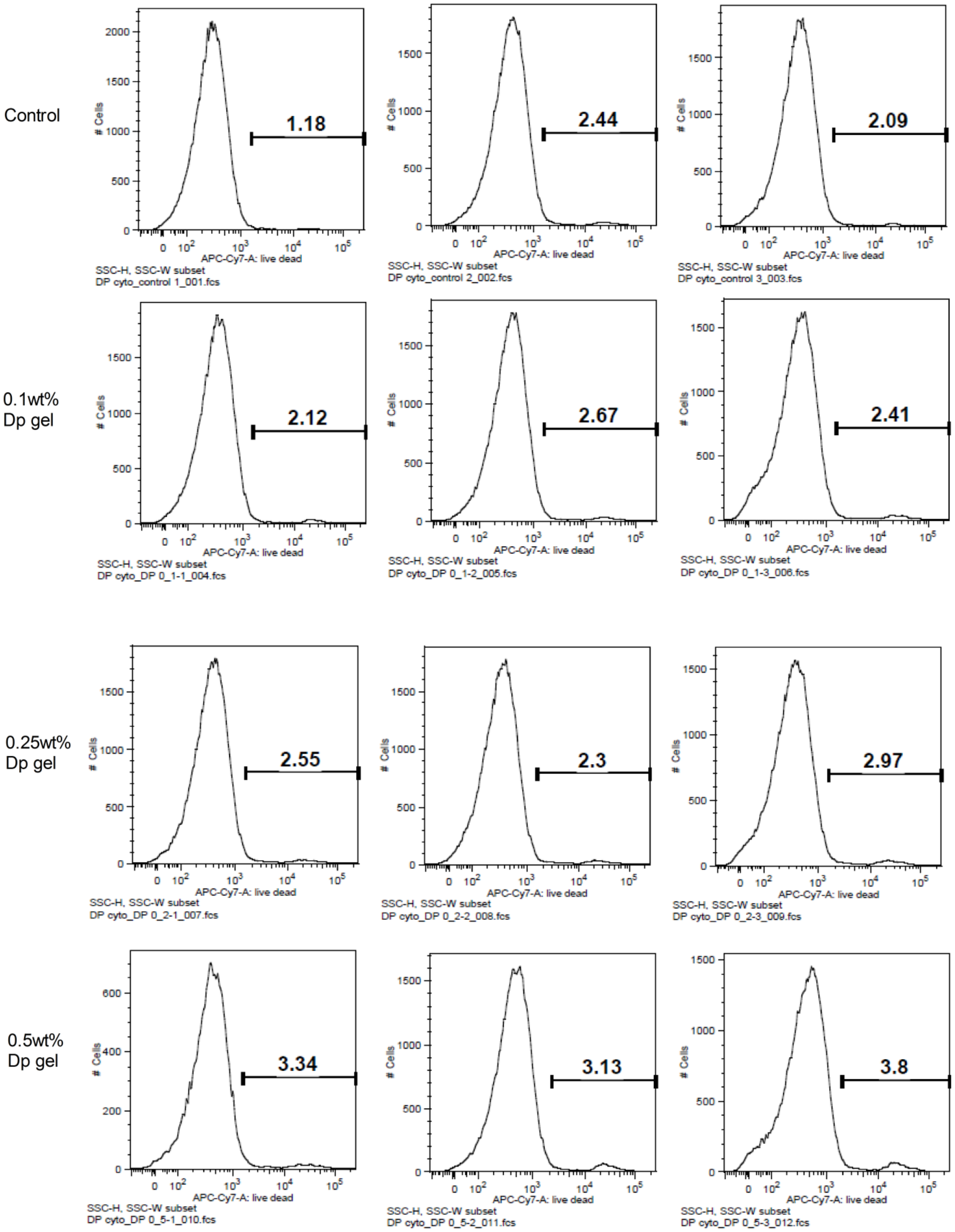

Control
0.1wt% Dp gel
0.25wt% Dp gel
0.5wt% Dp gel
1.18
2.44
2.09
2.12
2.67
2.41
2.55
2.3
2.97
3.34
3.13
3.8
# Cells
APC-Cy7-A: live dead
SSC-H, SSC-W subset
DP cyto_control 1_001.fcs
DP cyto_control 2_002.fcs
DP cyto_control 3_003.fcs
DP cyto_DP 0_1-1_004.fcs
DP cyto_DP 0_1-2_005.fcs
DP cyto_DP 0_1-3_006.fcs
DP cyto_DP 0_2-1_007.fcs
DP cyto_DP 0_2-2_008.fcs
DP cyto_DP 0_2-3_009.fcs
DP cyto_DP 0_5-1_010.fcs
DP cyto_DP 0_5-2_011.fcs
DP cyto_DP 0_5-3_012.fcs

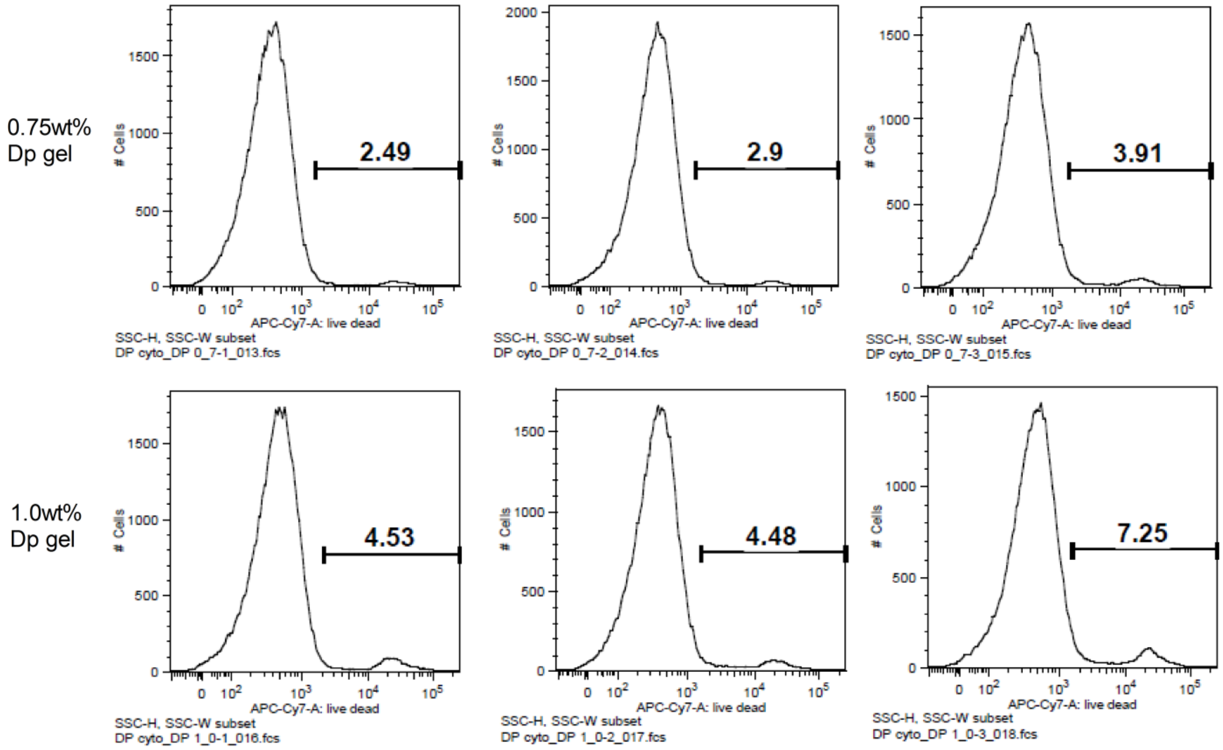


Figure S-11. Histograms showing cytotoxicity of HEK293T cells treated with Dp gels of different peptide concentration determined by live/dead assay using FACS analysis.

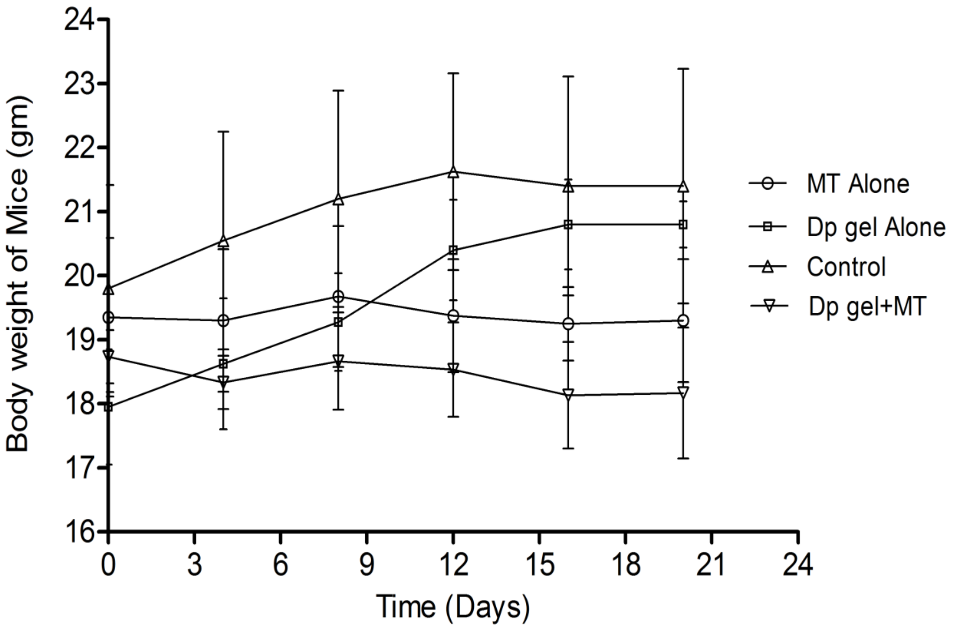


**Figure S-12.** Graph showing changes in body weight of different groups of mice treated with different formulations with time. Graph represents mean ± standard deviation (n=4).

**Analytical RPHPLC of LeuΔPhe at 280nm**

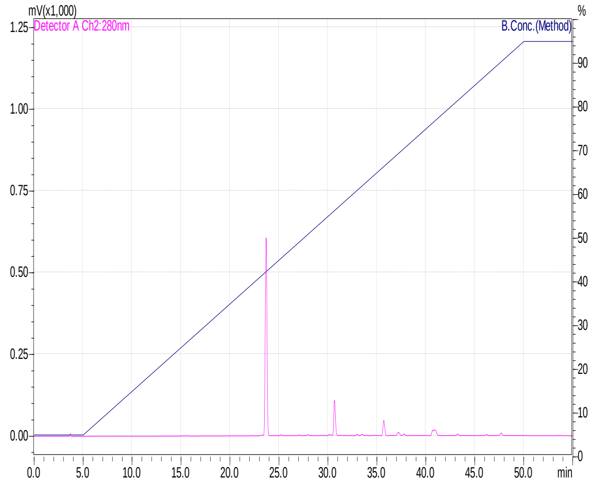


**ESI-MS profile of LeuΔPhe**

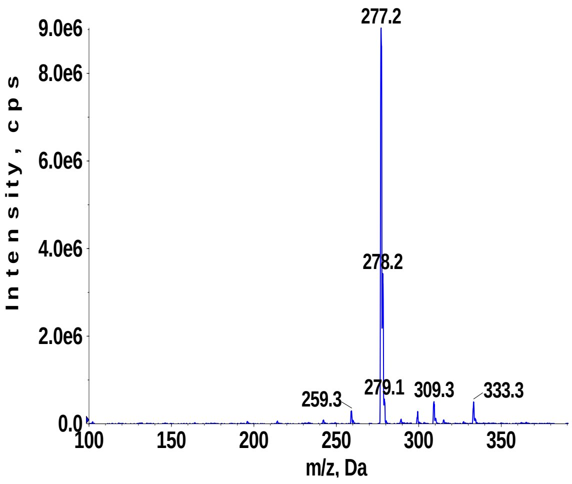


**Expected Mass: 276.35 Da**
**Observed Mass: 277.2Da**

**Figure S-13.** Analytical RP-HPLC and ESI-MS profiles LeuΔPhe**.**

## 3. Supplementary Tables:

**Table S1. List of ΔF (α, β-dehydrophenylalanine) containing dipeptides.**

| S.No. | Peptide Name | Peptide Sequence | Molecular Weight (Dalton) |
|---|---|---|---|
| 1. | Arginine-α,β-dehydrophenylalanine | RΔF | 319.38 |
| 2. | Leucine-α,β-dehydrophenylalanine | LΔF | 276.35 |
| 3. | Phenylalanine-α,β-dehydrophenylalanine | FΔF | 310.37 |
| 4. | Glutamic Acid-α,β-dehydrophenylalanine | EΔF | 292.31 |
| 5. | Threonine-α,β-dehydrophenylalanine | TΔF | 264.3 |
| 6. | Serine-α,β-dehydrophenylalanine | SΔF | 250.27 |
| 7. | Tyrosine-α,β-dehydrophenylalanine | YΔF | 326.37 |
| 8. | Aspartic Acid-α,β-dehydrophenylalanine | DΔF | 278.28 |
| 9. | Glutamine-α,β-dehydrophenylalanine | QΔF | 291.32 |
| 10. | Valine-α,β-dehydrophenylalanine | VΔF | 262.32 |
| 11. | Tryptophan-α,β-dehydrophenylalanine | WΔF | 349.41 |
| 12. | Glycine-α,β-dehydrophenylalanine | GΔF | 220.24 |
| 13. | Proline-α,β-dehydrophenylalanine | PΔF | 260.31 |
| 14. | Asparagine-α,β-dehydrophenylalanine | NΔF | 277.3 |
| 15. | Isoleucine-α,β-dehydrophenylalanine | IΔF | 276.35 |
| 16. | Methionine-α,β-dehydrophenylalanine | MΔF | 294.39 |

**Table S2. Clog P, net charge, molecular mass and D values of drugs.**

| S.No. | Drugs | Molecular mass (Da) | Clog P | Net Charge | D values ($10^{-10}$ $m^2/s$) |
|---|---|---|---|---|---|
| 1. | Neomycin | 614.31 | -7.8 | 6 | 1.7 |
| 2. | Streptomycin | 581.26 | -6.4 | 3 | 2.9 |
| 3. | Mitoxantrone | 444.2 | -3.1 | 2 | 3.4 |
| 4. | Spectinomycin | 332.34 | -2.3 | 2 | 4.1 |
| 5. | Cisplatin | 296.9 | -2.19 | 0 | 4.8 |
| 6. | Isoniazid | 137.05 | -0.7 | 0 | 6 |
| 7. | Fluconazole | 306.1 | 0.4 | 0 | 0.09 |
| 8. | Amphotericin B | 923.48 | 0.8 | 0 | 0.07 |
| 9. | Vitamin $B_{12}$ | 1355.57 | 1.87 | 2 | 0.04 |
| 10. | Rifampicin | 822.4 | 2.7 | 1 | 0.03 |
| 11. | Paclitaxol | 853.33 | 3 | 0 | 0.02 |
| 12. | Curcumin | 368.37 | 3.2 | 0 | 0.01 |
